# Down-regulation of UHRF1, associated with re-expression of tumor suppressor genes, is a common feature of natural compounds exhibiting anti-cancer properties

**DOI:** 10.1186/1756-9966-30-41

**Published:** 2011-04-15

**Authors:** Mahmoud Alhosin, Tanveer Sharif, Marc Mousli, Nelly Etienne-Selloum, Guy Fuhrmann, Valérie B Schini-Kerth, Christian Bronner

**Affiliations:** 1 CNRS UMR 7213 Laboratoire de Biophotonique et Pharmacologie, Université de Strasbourg, Faculté de Pharmacie, 74 route du Rhin, 67401 Illkirch, France

## Abstract

Over-expressed in numerous cancers, Ubiquitin-like containing PHD Ring Finger 1 (UHRF1, also known as ICBP90 or Np95*) *is characterized by a SRA domain (Set and Ring Associated) which is found only in the UHRF family. UHRF1 constitutes a complex with histone deacetylase 1 (HDAC1) and DNA methyltransferase 1 (DNMT1) via its SRA domain and represses the expression of several tumour suppressor genes (TSGs) including *p16*^*INK4A*^, *hMLH1, BRCA1 *and *RB1*. Conversely, UHRF1 is regulated by other TSGs such as *p53 *and *p73*. UHRF1 is hypothetically involved in a macro-molecular protein complex called "ECREM" for "Epigenetic Code Replication Machinery". This complex would be able to duplicate the epigenetic code by acting at the DNA replication fork and by activating the right enzymatic activity at the right moment. There are increasing evidence that UHRF1 is the conductor of this replication process by ensuring the crosstalk between DNA methylation and histone modifications via the SRA and Tandem Tudor Domains, respectively. This cross-talk allows cancer cells to maintain the repression of TSGs during cell proliferation. Several studies showed that down-regulation of UHRF1 expression in cancer cells by natural pharmacological active compounds, favors enhanced expression or re-expression of TSGs, suppresses cell growth and induces apoptosis. This suggests that hindering UHRF1 to exert its role in the duplication of the methylation patterns (DNA + histones) is responsible for inducing apoptosis. In this review, we present UHRF1 expression as a target of several natural products and we discuss their underlying molecular mechanisms and benefits for chemoprevention and chemotherapy.

## 1. Introduction

Cancer is one of the main causes of death among Westernized countries and is principally due to environmental risk factors, including diet [1]. It is caused by a series of genetic and epigenetic abnormalities that induce the activation of oncogenes and/or the inactivation of tumour suppressor genes (TSGs) [2,3]. For instance, colorectal cancer is known to be a consequence of successive genetic and epigenetic changes [4,5]. Indeed, an aberrant promoter hypermethylation of the *hMLH1 *gene (Human Mutant L homologue 1) is a potential major cause of colon carcinogenesis suggesting that an epigenetic mechanism is underlying tumorogenesis [6]. The term epigenetic is defined as heritable modification in gene expression without any variation in the DNA sequence [2,3,7,8]. DNA methylation and histone post-translational changes are the two main hallmarks of the epigenetic process. Unlike the genetic abnormalities which are irreversible, epigenetic alterations could be reversible making them as interesting therapeutic targets. Epigenetic regulation of gene expression is particularly sensitive to environmental conditions, including diet [9]. A few examples clearly demonstrate that dietary behaviours can affect the future health of subsequent generations, by increasing the risk of cardio-metabolic diseases such as diabetes mellitus, hypertension and obesity [9].

Concerning cancer and transgenerational epigenetic effect of diets, in terms of increased risk, no evidence has so far yet been reported. However, cancerogenesis is now recognised as being the result of profound dietary-influenced epigenetic modifications, among which hypermethylation of the promoters of several TSGs occupies a main place [3,10]. Reversing promoter methylation of silenced tumor suppressor genes represents a current challenge for anti-cancer therapy.

### 2. DNA methylation and histone modifications in cancer

In mammalians, DNA methylation is the most widely studied epigenetic modification. It is mediated by a family of DNA methyltransferases (DNMTs) that transfer a methyl group (CH3) from the methyl donor S-adenosylmethionine at the carbon in the fifth position of cytosine in CpG dinucleotides [11,12]. This family includes several members, *i.e*. DNMT1, DNMT3A and DNMT3B [13]. DNMT2 and DNMT3L have very little methyltransferase activity and will not be discussed here [13]. While about 80% of isolated CpG sites in the genome are methylated, the « CpG islands » (CpG-rich short regions of DNA) are usually unmethylated [14]. Exceptions are some CpG island promoters which remain methylated during development. X-chromosome inactivation and imprinted genes are the two known examples of these exceptions [15]. In cancer cells, in contrast to genome-wide hypomethylation which increases genomic instability and activates growth-promoting genes (proto-oncogenes), promoters of tumour suppressor genes are frequently hypermethylated and this contributes to carcinogenesis [16]. Various TSGs are silenced in cancer cells by promoter hypermethylation such as *RB1*, *H1C1 *(Hypermethylated In Cancer 1), *p16*^*INK4A*^, *MLH1 *(Human Mutant L homologue 1), *BRCA1 *(BReast CAncer 1) and *p73 *[17-23]. While the capacity of CpG island hypermethylation to induce TSGs silencing is well studied, the mechanism by which these TSGs are specifically targeted is still unclear. One hypothesis is that CpG island hypermethylation of TSGs is driven by a mechanism involving unknown DNA binding factors that selectively recruit DNMT1 to the promoters of TSGs which will lead to pathological hypermethylation and subsequently to unpaired apoptosis.

Many evidences of the crosstalk between DNA methylation and histone modifications have been reported [24,25]. The most important histones modifications, having effects on gene expression, are located on histone H3 and histone H4 [26]. One of them, that is known to have a gene silencing role and to have a strong relationship with DNA methylation, is the di- or tri-methylation of lysine 9 of histone 3 (H3K9me2 or H3K9me3). But methylation on the same histone on lysine 4 (H3K4me) is related to gene activation. All these modifications are catalysed by a broad variety of specific enzymes, some of which can catalyse the same reaction but at different location in the nucleus, *i.e.*, heterochromatin or euchromatin [26].

Histones undergo specific changes in their acetylation and methylation degrees during cancerogenesis [27]. Both deacetylation of H4K16 and accumulation of H3K9me2 are found on many repressed genes, including TSGs [27,28]. These modifications are mediated by HDACs (histone deacetylases) and G9a (histone 3 methyltransferase) respectively. HDACs are often over-expressed in various types of cancer such as renal cancer [29] or gastric cancer [30] and have become essential targets for anticancer therapy. G9a is co-localized near the methylated promoters of numerous genes in cancer cells [31]. Interestingly, it has been found that the inhibition of G9a is sufficient to induce a reactivation of TSGs [32]. Therefore, over-expression of enzymes catalysing histone modifications (epigenetic writers), might be one explanation for the occurrence of altered epigenetic marks found in cancer.

**Figure 1:**
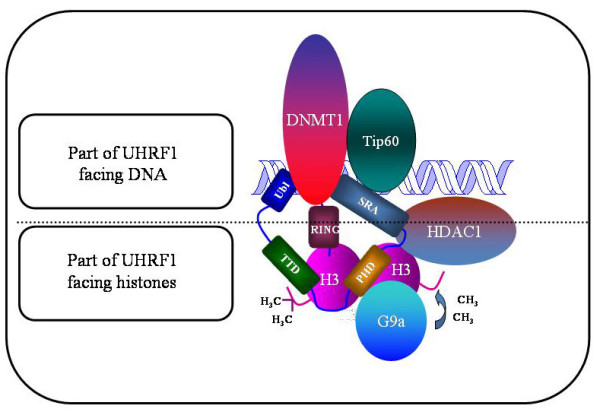
**Schematic representation of UHRF1 with the structural domains facing either DNA or histones**. Abbreviation: UBL, Ubiquitin-like domain; TTD, cryptic Tandem Tudor Domain; PHD, Plant Homeo Domain; SRA, Set and Ring Associated; RING, Really Interesting New Gene. The major partners of UHRF1, namely Tat-Interactive Protein of 60 kDA (Tip60), DNA methyltransferase 1 (DNMT1), histone methyltransferase G9a (G9a) and Histone DeAcetylase (HDAC1) are also depicted.

There is increasing evidence that Ubiquitin-like containing PHD Ring Finger 1 (UHRF1, also known as ICBP90 or Np95) plays a fundamental role in these processes by being involved in DNA methylation, histone methylation, histone acetylation, cell proliferation and apoptosis. This is due to the fact that UHRF1 possesses several domains (Figure 1) able to read both DNA methylation and histone methylation, thus, physically linking these two epigenetic marks [26,33,34].

### 3. UHRF1 and DNA methylation and histone modifications patterns

UHRF1, a putative oncogenic factor, is over-expressed in numerous cancers [35,36] and has been suggested to be an important biomarker to discriminate between cervical high-grade and low-grade cancer lesions [37]. Another study has highlighted the efficiency of UHRF1 as a marker to differentially diagnose pancreatic adenocarcinoma, chronic pancreatitis and normal pancreas [38]. UHRF1 over-expression was also found in bladder cancer and the intensity of its over-expression appears to be related to the stage of the cancer [39], suggesting that the presence of UHRF1 in urine sediment or surgical specimens could be a useful diagnostic marker and may improve the diagnosis of the bladder cancer. Recently, UHRF1's overpression has also been described in lung cancer cells, particularly in non-adenocarcinomas [40]. This alteration in UHRF1 expression could be linked to the degree of the lung cancer aggressiveness and was detectable in half of the patients in an early pathological stage. This suggests therefore that UHRF1 could be a novel diagnostic tool for lung cancer [40]. Altogether, these clinical studies show that immuno-histochemical staining of UHRF1 may improve the specificity and sensitivity of current tests for cancer diagnosis. These studies also emphasize that over-expression of UHRF1 might be involved in the establishment of aberrant histone code and altered DNA methylation patterns. The consequences of UHRF1 over-expression are cell contact inhibition loss [41] and inhibition of TSGs expression, such as *CDKN2A *and *RASSF1 *[42]. Furthermore, very recently, it was shown that UHRF1 down-regulation in p53 containing and deficient cancer cells induced cell cycle arrest in G2/M and caspase-8-dependent apoptosis [43]. This is consistent with previous studies showing that down-regulation of UHRF1 leads to cell growth inhibition [44-46].

UHRF1 is characterized by the presence of several structural domains, some facing DNA and others facing histones (Figure 1). Among them, one of the most amazing domain is undoubtedly the SRA domain (Set and Ring Associated) which, in vertebrates, is found only in the UHRF family [35]. Thanks to this domain, UHRF1 interacts with histone deacetylase 1 (HDAC1) and can bind to methylated promoter regions of various TSGs, including *p16*^*INK4A *^and *p14*^*ARF *^[44]. Moreover, we have shown that UHRF1, via the SRA domain, associates with DNA methyltransferase 1 (DNMT1) to form a couple cooperating in the duplication of the DNA methylation patterns but other domains of UHRF1 could also be involved [26,47-49]. The mechanism of DNA methylation pattern duplication, involves the SRA domain which is able to detect the hemi-methylated state of the DNA that occurs after the synthesis of the new DNA strand [50-52]. This domain behaves as a "hand" with a palm which holds the methylated cytosine, after that two "fingers" have flipped the methylated cytosine out from the DNA helix into the major DNA groove. The flipped methylated cytosine allows UHRF1 to be anchored at the hemi-methylated site to give the time necessary for DNMT1 to methylate the newly synthesized DNA strand [26,53], thus ensuring the maintenance of the DNA methylation patterns through successive cell divisions. Altogether, these observations show that immediately after DNA replication which generates hemi-methylated strands, UHRF1 is recruited with DNMT1 and/or likely DNMT3a and DNMT3b, in order to perpetuate gene repression, and particularly that of TSGs in cancer cells.

Recently, two novel and interesting partners of UHRF1, namely Tip60 (Tat-Interactive Protein) and HAUSP (Herpes virus-Associated Ubiquitin Specific Protease) have been identified [54,55]. Indeed, we showed that Tip60 is present in the same macromolecular complex as UHRF1, DNMT1, and HDAC1. Tip60 is a histone acetyltransferase with specificity toward lysine 5 of histone H2A (H2AK5) [54]. Interestingly, we observed that UHRF1 down-regulation correlated with an increase in Tip60 expression, which was associated with a decrease of acetylated H2AK5, suggesting that Tip60 requires UHRF1 for H2AK5 acetylation [54]. This mark could be involved in the epigenetic silencing of TSGs, but this possibility requires further investigations. The other studies reported that through an acetylation-dependent process UHRF1/Tip60 acts as destroyers of DNMT1 whereas HDAC1/HAUSP act as protectors for DNMT1 [55-57]. The paradigm resulting from this study additionally supports the idea of the existence of a macromolecular complex involved in the duplication of the epigenetic code that is capable of self regulation through external signals [57]. This complex is able to duplicate the epigenetic code after DNA replication and thus, allows cancer cells to maintain the repression of TSGs, including for instance *BRCA1 *and *p16*^*INK4A *^[49,58]. Indeed, it has been reported that UHRF1 is responsible for the repression of *BRCA1 *gene in sporadic breast cancer through DNA methylation, by recruiting DNMT1, and histone deacetylation or methylation, by recruiting HDAC1, or G9a, respectively [58]. As a platform protein, UHRF1 is expected to be the major conductor of the epigenetic orchestra by using various executors to facilitate the conservation of the silencing marks, especially those concerning TSGs repression in the cancer cells. Thus, targeting this epigenetic conductor may be a new promising approach for anticancer therapy.

Until today, only the two key partners of UHRF1 (DNMT1 and HDAC1) are targeted therapeutically. Indeed, two large families of specific inhibitors of DNMT1 (DNMTi) and HDAC1 (HDACi) are commercially available but which efficiency in solid tumors is often questioned [59,60]. The current challenge is therefore to find new targets which will enable to treat more efficiently cancer, with lower toxicity and more specificity to reduce the side effects of these chemical compounds. Considering that DNMT1 and probably HDAC1 require UHRF1 to fully exert their effects, inhibiting the UHRF1 activity or expression would theoretically mimic the cumulative effects of HDAC1 and DNMT1 inhibitors and thus would be highly efficient, especially in solid tumors in which DNMTs are particularly less active.

### 4. Targeting UHRF1 abundance by natural compounds

**Figure 2:**
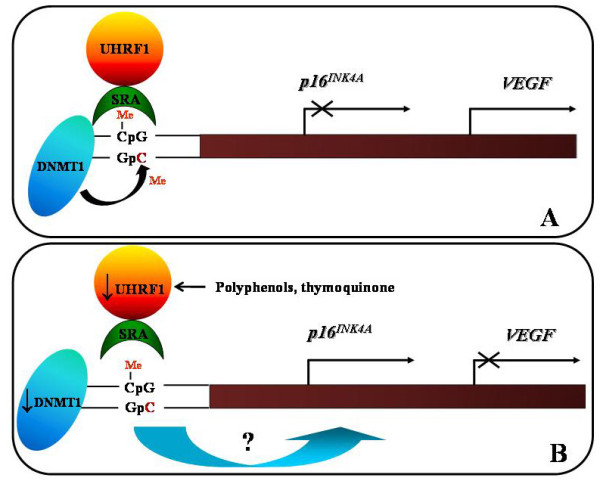
**Schematic model of the role of UHRF1/DNMT1 complex in the regulation of *p16***^***INK4A***^** and *VEGF *gene expressions**. **A**. When the SRA domain of UHRF1 meets hemi-methylated DNA present in the *p16*^*INK4A *^promoter, UHRF1 acts as a guide for DNMT1 to methylate the complementary DNA strand. Subsequently a *p16*^*INK4A *^gene repression and *VEGF *gene activation are maintained on the DNA daughter strands, *i.e.*, in the daughter cancer cells. **B**. The UHRF1 down-regulation, by natural compounds such as TQ or polyphenols, induces the DNMT1 abundance decrease, that is accompanied by a *p16*^*INK4A *^gene re-expression and a down-regulation of *VEGF *gene expression.

Targeting UHRF1 abundance and/or UHRF1's enzymatic activity would have application in several types of cancer. UHRF1 is essential for cell proliferation and therefore, to our opinion it would be more rational to target cancer types in which UHRF1 is actually found in high abundance, *i.e.*, over-expressed. UHRF1 has been reported to be over-expressed in various cancers such as breast, bladder, kidney, lung, prostate, cervical, and pancreatic cancers, as well as in astrocytomas and glioblastoma [35,40,61]. The anticancer strategic idea would be not to completely inhibit UHRF1 expression considering that UHRF1 is also necessary for non cancerous to proliferate [44,62,63], hence, for instance, for physiologic tissue regeneration. Thus, to consolidate the anti-UHRF1 therapeutic interest, it would be interesting to show that diminishing but not abolishing UHRF1's expression by chronic treatment of natural compound is sufficient for re-expression of silenced tumor suppressor genes. An ideal property for future natural compounds as anti-cancer drugs, would be that cancer cells but not normal cells are affected by them in order to undergo apoptosis via an UHRF1 down-regulation. Targeting UHRF1 is particularly interesting because this protein regulates the G1/S transition [47-49,62,63]. The arrest at G1/S checkpoint is mediated by the action of the tumor suppressor gene *p53 *or its functional homologue *p73 *[64,65]. Recent years have seen a dramatic progress in understanding mechanisms that regulate the cell division. In this context, we and other groups have shown that UHRF1 is essential for G1/S transition [63]. Loss of p53 activity, as a result of genetic mutations or epigenetic alterations in cancer, prevents G1/S checkpoints. DNA damage induces a *p53 *or *p73 *up-regulation (in p53-deficient cells) that activates the expression of *p21*^*cip/waf *^or *p16*^*INK4A*^, resulting in cell cycle arrest at G1/S transition [65,66]. We have shown that UHRF1 represses the expression of tumour suppressor genes such as *p16*^*INK4A *^&*RB1 *leading to a down-regulation of the Vascular Endothelial Growth Factor (VEGF, Figure 2A) [49] and by a feedback mechanism, UHRF1 may be regulated by other tumour suppressor genes such as *p53 *and *p73 *products [46,67]. This suggests that the appearance of genetic and/or epigenetic abnormalities of TSGs including *p53 *and *p73 *genes, in various human cancers would be an explanation for the observed UHRF1 over-expression. Since UHRF1 controls the duplication of the epigenetic code after DNA replication, the inability of p53 and P73 to down-regulate UHRF1, allows the daughter cancer cells to maintain the repression of tumour suppressor genes observed in the mother cancer cell [26,68].

Over the last millenium, herbal products have been commonly used for prevention and treatment of various diseases including cancer [69-71]. One of these natural products is curcumin which has potent anti-cancer properties in experimental systems. Curcumin is consumed in high quantities in Asian countries and epidemiological studies have attributed the lower rate of colon cancer in these countries to its consumption [72]. Green tea is also widely consumed in Asia countries. This natural product, which is rich in polyphenols, has been shown to significantly decrease the risk of breast and ovarian cancers in women in Asian countries [73]. Black seed (*nigella sativia*) belongs to the *Ranunculaceae *family which grows in the Mediterranean sea and Western Asia countries, including Pakistan, India and China [74]. This plant is used in traditional folk medicine for the prevention and the treatment of numerous diseases such as eczema, cough, bacterial and viral infections, hypertension and diabetes [75]. The chemotherapeutic and chemopreventive activities of black cumin oil are attributed to thymoquinone (TQ). Several *in vitro *and *in vivo *studies have shown that TQ has potent cytotoxic and genotoxic activities on a wide range of cancer cells [76-80]. TQ exerts its anti-cancer effects by inhibiting cell proliferation, arresting cell cycle progression and inducing subsequently apoptosis by p53- dependent or -independent pathways. By using the acute lymphoblastic leukemia jurkat cell model (p53 mutated cell line), we have demonstrated that TQ triggers apoptosis through the production of reactive oxygen species (ROS) and the activation of the *p73 *gene [67]. This tumor suppressor gene seems to act as a cellular gatekeeper by preventing the proliferation of TQ-exposed Jurkat cells [67]. Obviously, the observed p73 activation triggers G1 cell cycle arrest and apoptosis. Interestingly, a transient TQ concentration-dependent up-regulation of caspase 3 cleaved subunits was also observed, suggesting that TQ exerts its apoptotic activity through a p73-dependent caspase-dependent cell death pathway. Consistently with our study, it was recently reported that catechin, a natural polyphenolic compound, induces apoptosis, in a similar way as does TQ, by its ability to increase the expression of pro-apoptotic genes such as caspase-3, -8, and -9 and p53 [81]. Interestingly, our study also showed that TQ down-regulated UHRF1, DNMT1 and HDAC1 expressions [67]. We determined that p73 was responsible for UHRF1 down-regulation through a caspase-3 dependent process. A subsequent study allowed us to propose that down-regulation of phosphodiesterase 1A (PDE1A), a modulator of cAMP and cGMP cyclic nucleotides, could be the key event to explain the TQ-induced down-regulation of UHRF1 and the occurrence of apoptosis [82]. All these findings showed for the first time that a natural compound induces apoptosis by acting on the epigenetic integrator UHRF1 through a p73-dependent mitochondrial pathway.

**Figure 3:**
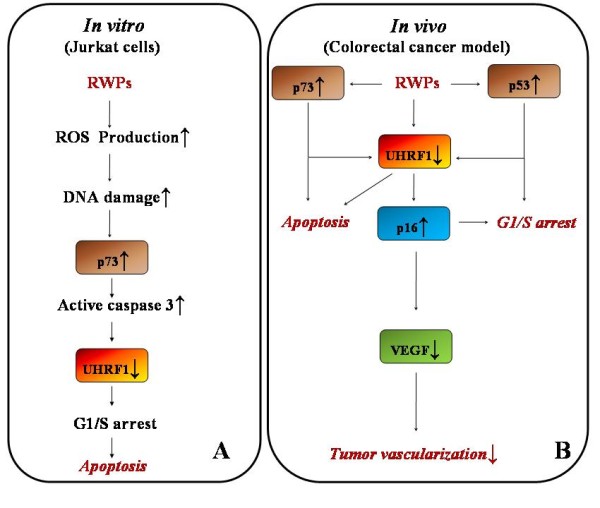
**Schematic representation of RWPs-induced apoptosis involving p73 and  UHRF1 deregulation in Jurkat cells and in an in vivo colorectal cancer model.**  A. Schematic representation of RWPs-induced apoptosis involving p73 and UHRF1 deregulation in Jurkat cells. RWPs triggers production of reactive oxygen species (ROS) and putatively DNA damage. The activation of the p73 gene results in enhanced caspase 3 level inducing UHRF1 decrease with subsequent G1/S arrest and apoptosis. **B**. The pathway involved *in vivo *is similar to that observed in Jurkat cells by involving a down-regulation of UHRF1 with subsequent increase of *p16*^*INK4A *^gene expression. The down-regulation of UHRF1 is probably driven by p53 and/or p53. This is leading to an inhibition of tumor vascularization as a consequence of the down-regulation of the *VEGF *gene expression.

Epidemiological studies report that diets rich in fruits and vegetables reduce the rate of cancer mortality [83-87]. The beneficial effects of these diets are attributed, at least partly, to polyphenols which have been described to have *in vitro *and *in vivo *anti-tumoral properties in several types of cancer cells [88-90]. Red wine is one of the most abundant source of polyphenols and represents an important occidental dietary component. In recent years, epidemiological studies have demonstrated the cancer chemopreventive effects of red wine polyphenols (RWPs) [91,92]. In this context, we found that a whole extract of RWPs dose-dependently inhibits the proliferation of various cancer cell lines, including the acute lymphoblastic leukemia Jurkat and the P19 teratocarcinoma cell lines [93,94]. This growth inhibition was correlated with an arrest of cell cycle progression in G1 and to subsequent apoptosis. Further investigations allowed us to observe that RWPs-exposed leukemia cells exhibit a sharp increase of p73 level associated with a significant decrease in UHRF1 expression, in agreement with Alhosin *et al.*, [67]. These findings indicate, therefore, that RWPs extract likely triggers cell cycle arrest and apoptosis by targeting UHRF1 through a p73-dependent pathway and a ROS-dependent process. Interestingly we have also observed that a RWPs extract significantly increased the formation of ROS (Figure 3A). Consistently, it has been recently shown that saikosaponins sensitize cancer cells to cisplatin through ROS-mediated apoptosis, and the combination of saikosaponins with cisplatin could be an effective therapeutic strategy [95].

An *in vivo *study has demonstrated that RWPs administrated with diet to rats inhibited azoxymethane-induced colon carcinogenesis [96], but the involved molecular mechanism remains unclear. Thus, to confirm *in vivo *the pathways involved in the protective effects of RWPs, we used a mouse model of colorectal cancer, by sub-cutaneously injecting C26 cells [97]. By using micro-angiography and immunohistochemistry approaches, we showed that regular consumption of RWPs in the drinking water decreased C26 tumour vascularization in BALB/C mice as a consequence of decreased expression of major proangiogenic factors including VEGF, matrix metalloproteinase 2 and 9, and cyclooxygenase-2 [97]. The RWPs-induced down-regulation of proangiogenic factors was associated with an activation of various TSGs such as *p53, p73, p16*^*INK4A *^and the cell cycle regulator *p21*^*Waf1/Cip1*^. Interestingly, a strong immunostaining for UHRF1 was observed in the tumours from the control group, whereas low staining was found in those from RWPs-treated group. These results suggest a specific role of this epigenetic actor in the progression of colorectal tumor. Therefore, UHRF1 abundance is likely a preferred target of RWPs in C26 cells-induced tumorigenesis mouse model. However, the precise mechanism by which RWPs induce the up-regulation of TSGs in colorectal cancer models is presently unclear. Recently, it has been shown that apple polyphenols has potent DNA demethylation activity in colorectal cancers by reducing DNMT1 expression with a subsequent activation of TSGs such as *hMLH1*, *p14*^*ARF *^and *p16*^*INK4A*^. These genes are known to be silenced through their promoter hypermethylation in colorectal cancers [98]. Consistently with this, it was recently shown that the polyphenol epigallocatechin gallate allows re-expression of *p16*^*INK4A *^and *p21*^*Waf1/Cip1 *^through a DNA demethylation dependent process probably involving a down-regulation of DNMT1 [99]. In agreement with our previous studies [49,67], we propose two mechanisms targeting UHRF1 and underlying the antitumoral activities of RWPs in colorectal cancer. First, considering that UHRF1 binds to methylated promoters of TSGs, *i.e., p16*^*INK4A *^[44], and that UHRF1 interacts with DNMT1 and regulates its expression [49], it is likely that the RWPs-induced down-regulation of UHRF1, with subsequent decrease of DNMT1, could be involved in the demethylation of the *p16*^*INK4A *^promoter (Figure 2B). Second, RWPs could trigger cell cycle arrest and apoptosis in colorectal cancer by activation of *p53 *and *p73 *which are negative upstream regulators of UHRF1 [46,67]. These findings suggest that RWPs exert their antitumoral activities in colorectal cancer through a mechanism of feedback control involving TSGs and UHRF1 (Figure 3B). Thus, targeting UHRF1 by natural compounds could be an interesting way to prevent and/or to treat colorectal cancers.

Combination of HDACs and DNMT1 inhibitors exhibits synergic anti-neoplasic effect for different types of cancer [100-103]. A phase I pilot study showed that chronic intake of black raspberries by patients suffering from colorectal cancers leads to down-regulation of DNMT1 and re-expression of TSGs through a DNA demethylating process [104]. This suggests that a therapeutically-induced inhibition of UHRF1 activity or expression could prevent the action of its preferred partners, HDAC1 and DNMT1, leading to a re-expression of the tumour suppressor genes *p16*^*INK4A *^and thus allowing the cancer cells to undergo apoptosis.

## Conclusion

**Figure 4:**
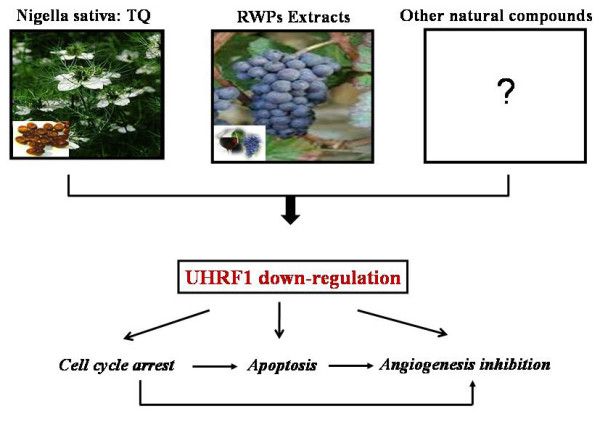
**Summary of the effects of natural products such as TQ and RWPs**. These compounds are putative "regulators" of the epigenetic code inheritance, since they are able to target UHRF1 with a subsequent cell cycle arrest, apoptosis and tumor vascularization reduction. An open square containing a question mark, emphases the possibility that numerous other natural compounds can take the same pathways leading to apoptosis.

Natural compounds such as TQ, RWPs and potentially others (Figure 4) are triggering a series of events that involve cell cycle arrest, apoptosis and inhibition of angiogenesis, all under the control of UHRF1. UHRF1 is a key component of a macro-molecular complex including among others HDAC1, DNMT1, Tip60 and HAUSP, responsible for the epigenetic code duplication after DNA replication. UHRF1 behaves as a conductor in this replication by performing a crosstalk between DNA methylation and histone modifications. This allows cancer cells to maintain their pathologic repression of TSGs during cell proliferation. This review supports the paradigm that UHRF1 is a potential target for cancer prevention and therapy, since its repression may lead to the re-expression of TSGs, allowing cancer cells to undergo apoptosis. Natural anticancer products have been shown to suppress the expression of UHRF1. This suggests that these chemo-preventive and chemotherapeutic compounds potentially have the virtues to repair the "wrong" epigenetic code in cancer cells by targeting the epigenetic integrator UHRF1. It is very legitimate to propose that down-regulation of UHRF1 by natural compounds is a key event in their mechanism of action, considering that re-expression of tumor suppressor genes in cancer cells is dependent upon demethylation of their promoters and that UHRF1 is involved in the maintenance of DNA methylation patterns. These studies also highlight that UHRF1 and its partners are putative targets for the adaptation to environmental factors, such as diet. We also do not exclude that the behavior of the epigenetic code replication machinery, ECREM, might influence transgenerational message of environmental factors.

## Competing interests

The authors declare that they have no competing interests.

## Authors' contributions

MA and CB designed the review and drafted part of it. TS, MM, NES, GF and VBSK equally contributed to the writing the other part of the review. All authors read and approved the final manuscript.
